# Intraskeletal histovariability and skeletochronology in an ornithopod dinosaur from the Maestrazgo Basin (Teruel, Spain)

**DOI:** 10.1111/joa.14225

**Published:** 2025-01-28

**Authors:** Juan Maíllo, Jerome Hidalgo‐Sanz, José Manuel Gasca, José Ignacio Canudo, Miguel Moreno‐Azanza

**Affiliations:** ^1^ Departamento de Ciencias de la Tierra, Aragosaurus‐IUCA: Recursos Geológicos y Paleoambientes Universidad de Zaragoza Zaragoza Spain; ^2^ Departamento de Geología Universidad de Salamanca Salamanca Spain; ^3^ Department of Earth Sciences, GEOBIOTEC NOVA School of Science and Technology Caparica Portugal

**Keywords:** Iguanodontia, ontogeny, osteohistology, sexual maturity, three‐front model

## Abstract

Ornithopods are an extinct group of dinosaurs that were particularly abundant and diverse in the Cretaceous of the Iberian Peninsula, and whose abundance in the Maestrazgo Basin has allowed numerous taxa to be identified over the last decade. Many of these fossil remains are still taxonomically indeterminate and require a more detailed study on both a macroscopic and microscopic scale. In this contribution, an osteohistological analysis is carried out on a partial skeleton—composed of five incomplete vertebrae, two dorsal ribs, an ischium, a fibula, and a tibia—found in the province of Aliaga (Teruel, NE Spain). We identified a progressive slowdown in tissue apposition and a variation in the type of growth marks generated in every bone, allowing a more precise identification of the ontogenetic stage of the specimen as a subadult individual. The skeletochronological correlation between the different elements also suggests that the specimen reached sexual maturity at around seven years of age and died between nine and twelve years of age. Likewise, the usefulness of the three‐front model is proven for the first time in an ornithopod dinosaur, as a tool for analysing the histology expressed by the different bone elements of a single specimen and inferring their skeletochronological potential. Comparison with other ornithopod taxa reveals the great variability that each bone element shows depending on the taxon analysed, which prevents us from determining a single element suitable for studying the skeletochronology of any ornithopod taxon.

## Graphical abstract

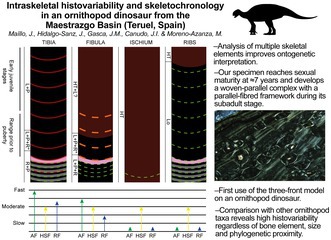

## 1 INTRODUCTION

Ornithopods are a diverse clade of ornithischian dinosaurs that stand out for their locomotor variations between bipedalism and quadrupedalism, their specialized phytophagous dentition, and their wide geographical distribution (e.g., Boyd, 2015; Maidment et al., 2013; Norman, 2004; Norman & Weishampel, 1985). The abundance of specimens belonging to different ontogenetic stages has made Ornithopoda one of the best‐studied clades of dinosaurs, allowing their growth dynamics, breeding strategies, and lifestyle adaptations to be reconstructed through the field of palaeohistology (Horner et al., 2000; Padian & Woodward, 2021; Woodward et al., 2015). They show a diverse array of growth strategies, including neoteny (Kitchener et al., 2019), adaptations to precocial or altricial behaviour (Hübner, 2012), and heterochronic development (Prondvai, 2014; Werning, 2012).

According to Werning (2012), two general growth dynamics emerge in function of their phylogenetic relationships and body size. The earliest‐diverging and smaller ornithopods develop a “basal growth syndrome”, where the histology reflects a pattern of initial fast growth followed by several years of sustained slower growth. By contrast, the deeply nested, generally larger iguanodontian ornithopods, develop a “syndrome of extended rapid growth” characterized by a rapid and constant growth rate until skeletal maturity is reached, at which time lamellar tissue develops abruptly.

Over the last decade, Werning's observations have been corroborated in some smaller early‐diverging ornithopods (Cruzado‐Caballero et al., 2019; Han et al., 2020; Prondvai, 2014; Waskow & Mateus, 2017; Woodward et al., 2018), in some of intermediate size (Bertozzo et al., 2024; Gates et al., 2018; Prondvai, 2014; Zheng et al., 2013), as well as in more nested hadrosaurids (Bertozzo et al., 2017; Fondevilla et al., 2018; Freedman Fowler & Horner, 2015; Gates et al., 2014; Prieto‐Márquez et al., 2016; Słowiak et al., 2020; Vanderven et al., 2014; Woodward, 2019; Woodward et al., 2015). Overall, the differentiation between the two phylogenetic extremes is discernible, but the transition between the two dynamics is still not well understood (Prondvai, 2014). A broader study of medium‐sized ornithopods, especially those around the base of Iguanodontia, seems to be the next step to address this issue.

This clade of ornithopods is notably abundant in the Lower Cretaceous of Europe, especially in Belgium, Britain and Spain (Bertozzo et al., 2024; Norman, 2013). The Iberian record of iguanodontian ornithopods is composed of some partial skeletons and numerous isolated remains, most of them from the Maestrazgo Basin in northeast Spain (e.g., Canudo et al., 2010; Gasulla et al., 2015; McDonald et al., 2012; Ruiz‐Omeñaca, 2011; Verdú et al., 2015). Within this group, non‐hadrosaurid styracosternans have traditionally been characterized by low anatomical disparity, which does not suggest a very broad taxonomic diversity (see Paul, 2008). However, during the last decade, new studies have identified numerous taxa that reflect a high and abundant diversity in the region (García‐Cobeña et al., 2022; Gasca et al., 2014a; Gasulla et al., 2015; Medrano‐Aguado et al., 2023; Santos‐Cubedo et al., 2021; Verdú et al., 2017). These discoveries have led to a conflictive and constant re‐evaluation of the group's phylogeny (Madzia et al., 2021; Rotatori et al., 2022) and highlighted the need to expand what is known of these animals and their defining characteristics.

As regards the microanatomical study of these Iberian taxa, various issues have been investigated in the last decade. These include histological development in perinatal or immature individuals (Perales‐Gogenola et al., 2019; Verdú et al., 2015), the insular modification of the predator‐prey relationship (Dieudonné et al., 2023), as well as cases of evolutionary convergence with distantly related taxa (Prieto‐Márquez & Sellés, 2023). Despite this recent increase in palaeohistological studies of the ornithopods of Spain, the abundance of material available and the wide taxonomic diversity require a significant effort to provide a global picture of the palaeohistology of this key clade in the Early Cretaceous ecosystems of Spain.

Here, we present an osteohistological analysis of a new specimen of an indeterminate iguanodontian ornithopod from the Maestrazgo Basin in Teruel Province. First, we analyse sections of axial and appendicular bones to identify the intraskeletal histovariability and how these differences can offer complementary information that enriches the interpretation of the histological history of the individual. To this end, we also propose for the first time the application of the three‐front model of Mitchell and Sander (2014) to an ornithopod dinosaur and compare its microanatomy with that of other ornithopods to provide more information on the growth dynamics proposed by Werning (2012). The final purpose of this work is to open a research line that reveals the microanatomy of the Iberian iguanodontians in a deep and systematic way.

## 2 GEOLOGICAL SETTING

**FIGURE 1:**
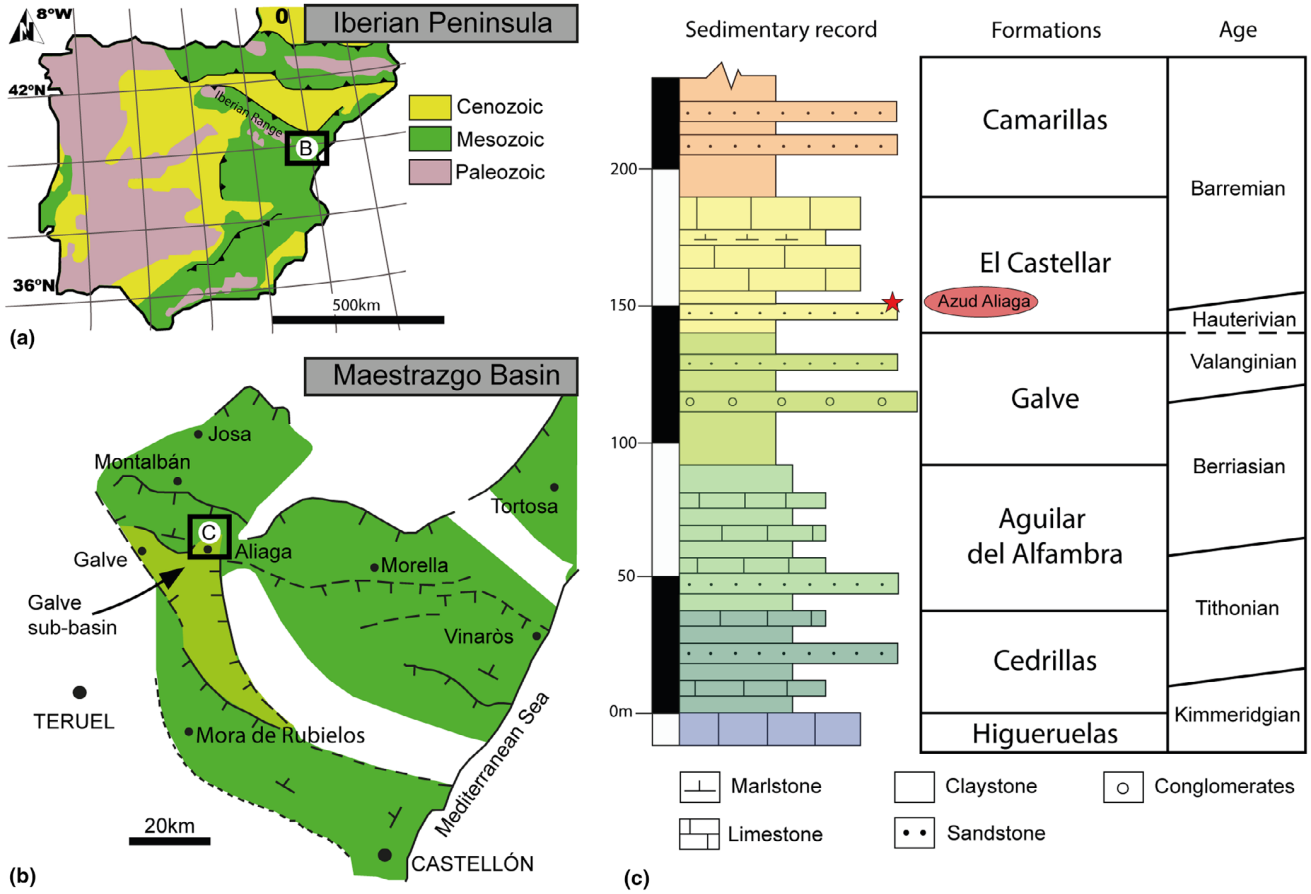
Geographical and geological setting of the Azud Aliaga fossil locality (Teruel Province, Spain). (a) Geological context of the Iberian Peninsula, modified from Salas et al. (2001). (b) Palaeogeographical location within the Maestrazgo Basin. (c) Synthetic log showing the stratigraphy (based on Aurell et al., 2016, 2019) of the Galve sub‐basin.

The material described here is from a small fossiliferous outcrop named Azud Aliaga, which is located in the municipality of Aliaga (Teruel Province, northeastern part of the Iberian Chain). The site is on a slope beside a road south of the town of Aliaga (Figure 1).

The Azud Aliaga fossil site is in the El Castellar Formation and is palaeogeographically located within the Galve sub‐basin, in the western part of the Maestrazgo Basin (Figure 1a,b). This basin forms part of the Iberian Rift System and developed during a rifting phase that started at the end of the Jurassic and lasted until the mid‐Albian (Álvaro et al., 1979; Liesa et al., 2018; Salas et al., 2001). The Upper Jurassic‐Lower Cretaceous stratigraphy of the Galve sub‐basin, summarized by Aurell et al. (2016), includes a syn‐rift sequence of predominantly continental‐transitional series corresponding to the Weald facies. The carbonate‐lutitic shallow‐lacustrine and palustrine facies represented by the El Castellar Formation across the entire Galve sub‐basin were deposited during the latest Hauterivian‐earliest Barremian (Aurell et al., 2016) according to the charophyte biozonation (Pérez‐Cano et al., 2022; Riveline et al., 1996).

The fossiliferous level of Azud Aliaga (Figure 1c) is situated in the lower part of the El Castellar Formation and comprises a grey lutitic layer overlying a 3 m‐thick layer of canaliform grey‐purple cross‐bedded sandstones. This facies association of channelled sandstones and grey lutites is characteristic of distal alluvial to palustrine conditions (Meléndez et al., 2009), as are usually seen in other vertebrate fossil sites of the El Castellar Formation (García‐Cobeña et al., 2022; Gasca et al., 2018).

## 3 MATERIALS AND METHODS

The fossil remains described here are associated remains of the skeleton of a single individual, including axial and appendicular bones. All these materials were collected with the corresponding permits under the local legislation and are housed in the Museo de Ciencias Naturales de la Universidad de Zaragoza (MPZ) (Canudo, 2018).

**FIGURE 2:**
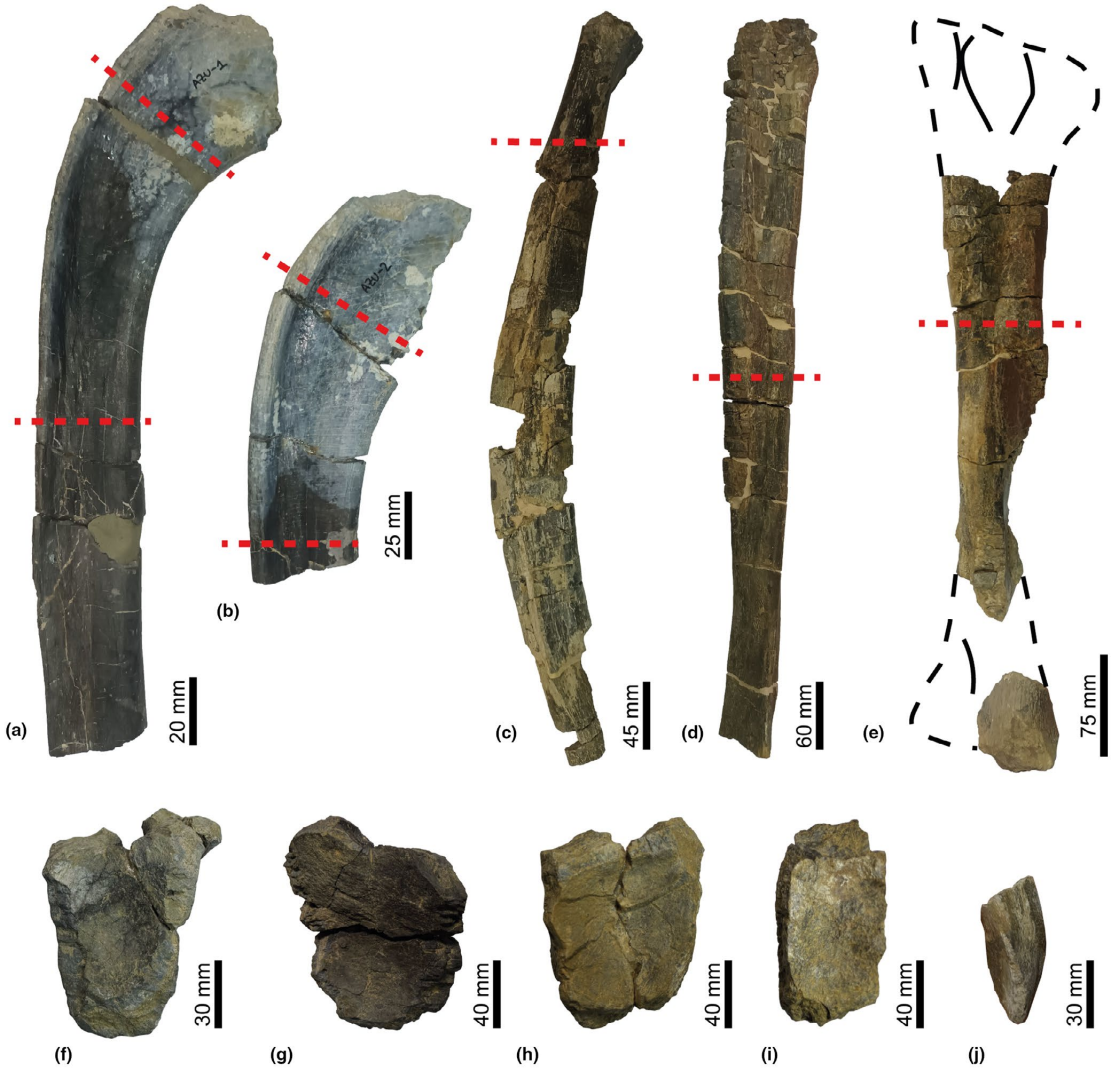
Iguanodontidae indet. from the Azud Aliaga site. In sample elements, the cutting levels are shown in red, and all orientations have the proximal side at the top and the distal side at the bottom. (a,b) Dorsal ribs MPZ 2024/92 and MPZ 2024/93 (posterior view). (c) Ischium MPZ 2024/94 (lateral view). (d) Fibula MPZ 2024/95 (medial view). (e) Tibia MPZ 2024/96 (lateral view). (f) Dorsal vertebra MPZ 2024/316 (anterior view). (g–i) Caudal vertebrae MPZ 2024/317, MPZ 2024/318 and MPZ 2024/319 (anterior view). (j) Prezygapophysis of an indeterminate caudal vertebra MPZ 2024/320 (dorsal view).

To undertake the skeletochronological analysis, thin sections of the diaphyseal areas of the preserved long bones, a fibula and a tibia, were selected. In addition, two dorsal ribs were also sectioned at proximal points under the tuberculum to capitalize on their skeletochronological potential (Waskow & Sander, 2014). Finally, a proximal fragment of a partially preserved ischium was also chosen, selecting a cutting point close to the flattest section of the bone and its ossification centre, a criterion previously applied in other pelvic bones (Hübner, 2012). Table 1 provides a complete list of all the material sampled for the palaeohistological study and its relevant details, and Figure 2a–e shows a view of the cut points in each bone. Figure 2 also includes a view of five incomplete vertebrae that will be considered later for the description and taxonomic designation of the specimen (Figure 2f–j).

**TABLE 1:** List of material included in the palaeohistological study.

Fossil bone ID	Element	Thin section ID	Side	Posteroanterior length at plane of sampling (mm)	Lateromedial length at plane of sampling (mm)
MPZ 2024/92	Dorsal rib	MPZ 2024/92‐L1	Left	16	50
MPZ 2024/92	Dorsal rib	MPZ 2024/92‐L2	Left	23	33
MPZ 2024/93	Dorsal rib	MPZ 2024/93‐L1	Left	26	46
MPZ 2024/93	Dorsal rib	MPZ 2024/93‐L2	Left	28	35
MPZ 2024/94	Ischium	MPZ 2024/94‐L1	Right	39	33
MPZ 2024/95	Fibula	MPZ 2024/95‐L1	Right	63	19
MPZ 2024/96	Tibia	MPZ 2024/96‐L1 & MPZ 2024/96‐L2	Right	75	81

To preserve the morphology and original dimension of each sample for future research, a virtual replica of each fossil was created by photogrammetry of each bone, using the Agisoft Metashape Professional software and a camera with a 48‐megapixel sensor and an angular lens with an f/1.8 aperture. Later, each bone was transversely cut at its selected point, following standard methods (Cerda et al., 2020; Chinsamy & Raath, 1992; Lamm, 2013). We prepared the thin sections at the Servicio General de Apoyo a la Investigación (SAI) of the Universidad de Zaragoza.

During the process, the sample MPZ 2024/96 (tibia) was divided into two smaller pieces due to its wide diameter, and in the case of MPZ 2024/92 and MPZ 2024/93 (dorsal ribs), additional cuts were made at the most distal points to verify the development of growth marks. In total, eight thin sections were prepared with an initial thickness of ≈100 μm (see Table 1). These sections were studied under transmitted light using an Olympus BX53M petrographic microscope housed at the facilities of the Instituto de Ciencias Ambientales de la Universidad de Zaragoza IUCA‐UNIZAR. To obtain singular images, OLYMPUS Stream Basic was executed with an Olympus DP27‐coupled camera. During photography, the observed area was sometimes moistened to increase the refraction index. The resulting images were processed using processing software, sometimes slightly altering the colour contrast and saturation levels to improve the visualization of certain structures.

Given the need to observe the samples also under polarized light in order to facilitate identification of structures such as secondary osteons or the degree of birefringence and the orientation of collagen fibres, the thin sections received a second polishing to ≈50–80 μm in thickness. This additional reduction allowed us to observe structures that could not be identified at the previous thickness under polarized light, but at the same time made it difficult to see others under transmitted light. Prior to this final polishing, all thin sections were digitized in the SAI with a Leica VM6 A microscope with an integrated 10‐megapixel camera at 100x magnification and using LAS X software.

Terminology for the histological markers follows de Buffrénil and Quilhac (2021), encouraging the use of “woven‐parallel complex” instead of “fibrolamellar tissue” (Prondvai et al., 2014). The term “multiple growth mark complex” is also used when several growth marks are identified in a closely grouped manner (Cullen et al., 2021). To discuss and compare the development of the tissues of each sample, the three‐front model proposed by Mitchell and Sander (2014) was used.

## 4 RESULTS

### 4.1 Systematic palaeontology

Dinosauria (Owen, 1842).

Ornithischia (Seeley, 1887).

Ornithopoda (Marsh, 1881).

Iguanodontia (Dollo, 1888).

Iguanodontia indet.

#### 4.1.1 Material

A partial associated skeleton, comprising five incomplete vertebrae (MPZ 2024/316 to 2024/320), two partial left dorsal ribs, a partial right ischium, a partial right fibula, and a partial right tibia (MPZ 2024/92 to 2024/96).

#### 4.1.2 Description

The recovered bones were disarticulated and show evidence of moderate weathering, with flaking in the form of longitudinal cracks due to subaerial exposure and subsequent recent alterations. All the bones show significant breakage, which is manifest mainly as fractures transverse to the long axis of the bones. These are also affected by the recent development of plant roots.

##### Vertebrae

MPZ 2024/316 is a fragmented dorsal vertebra that preserves part of the centrum, the base of the neural arch, and the initial part of both diapophyses. The margins of the articular facets are broken, and the posterior articular facet is only partially preserved. In anterior view, the neural canal can be seen, which is wider in its anterior part. In lateral view, the diapophyses appear in the upper part of the centrum, both of which are inclined in a proximal direction at approximately 45°. The neurocentral suture can be observed in lateral view and is also fused. The vertebra is slightly amphicoelous, with heart‐shaped articular faces that are wider dorsally.

MPZ 2024/317, MPZ 2024/318 and MPZ 2024/319 are part of a series of three mid‐caudal vertebrae, very close one to each other. They only preserve their amphicoelous to platycoelous vertebral centra, lacking the neural arches, the ventral facets for the haemal arches, and the margins of the articular faces due to breakage. Some of them have the lateral margins partially eroded. These centra have trapezoidal to square‐shaped articular faces, and all of them are amphicoelous. The articular faces are wider near to the neural arch—as in MPZ 2024/316—with inclined lateral margins forming a V‐shaped section. Finally, MPZ 2024/320 is a prezygapophysis from an indeterminately positioned caudal vertebra. Its medial surface is slightly depressed, and the lateral one forms a rounded bulge.

##### Dorsal ribs

MPZ 2024/92 is a proximal fragment of the shaft of a left anterior dorsal rib, whose exact position in the dorsal series cannot be specified. Its preserved length is 230 mm. The distal and proximal ends are broken, without preserving the capitulum and tuberculum. On the anterior surface of the shaft fragment, there is a longitudinal ridge close to the lateral edge of the rib, which moves slightly posteriorly. The posterior surface presents a concavity that decreases distally along the ramus of the rib, caused by the origin of a ridge in the lateral edge. The lateral surface is convex proximally and flattens distally. The development of these ridges induces a change in the section of the rib, passing from mace‐shaped in its proximal zone to reniform in the distal part.

MPZ 2024/93 is the proximal part of a left posterior dorsal rib, anatomically close to the rib described above. Its preserved length is 140 mm. Morphologically, it is similar to MPZ 2024/92, though slightly wider lateromedially and with its anterior and posterior ridges more marked.

##### Ischium

MPZ 2024/94 is a right ischium of which only a large part of the ischial branch has been preserved, with a length of 550 mm. In cross‐section, the major axis is 39 mm long and the minor axis measures 32 mm. It consists of an elongated bone that curves significantly. The curvature is greater in the most distal part of the ischial shaft, projecting anteriorly. It flattens distally, with an almost circular section in its proximal part, passing to elliptical in the distal zone of the bone. The dorsal margin of the ramus is rounded and tends to become more acute distally. On the ventral margin, there is a pointed ridge proximally that tends to soften distally. The medial surface of the ischium is concave, presenting a prominent groove in the proximal area, whereas the lateral surface is slightly convex and from it a prominent ridge emerges that develops along it.

##### Fibula

MPZ 2024/95 is a partial right fibula with a length of 655 mm, which does not preserve either epiphysis. It preserves a width of 79 mm in the proximal part and a width of 55 mm in the most distal zone. The fibular diaphysis is elongated, and in medial view, it is seen to be expanded anteroposteriorly, especially in its proximal half. In medial view, the proximal part of the bone hosts the articular facet for the tibia, which is concave in shape and deepens distally until the midpoint of the bone. The medial surface of the bone is flattened in its distal part and has sharp edges. In lateral view, a straight and rounded ridge is observed that arises from the anterior margin and develops in the distal half of the fibula and disappears towards the middle area of the shaft. The fibula changes in cross‐section from being eight‐shaped in the proximal half to subtriangular in the intermediate position and D‐shaped in the distal region.

##### Tibia

MPZ 2024/96 is a right tibia that preserves much of its shaft. Its proximal end is absent, whereas its distal end is incomplete and preserves part of the external malleolus. Its preserved length is 410 mm, and it is 100 mm wide in its proximal zone, changing to 55 mm in mid shaft. This elongated bone changes drastically in form along its length. It is compressed lateromedially and expanded anteroposteriorly near its proximal zone. In medial view, there appears a prominent U‐shaped groove in the proximal part of the bone that flattens towards the mid‐part of the tibia and develops a soft ridge that continues distally. In lateral view, the lateral surface can be seen to be more planar and slightly convex in the proximal part of the bone. This tibial shaft is reniform in cross‐section in the proximal part of the bone, changing to an almost D‐shaped section distally.

#### 4.1.3 Taxonomic discussion

In the Barremian ecosystems of the Maestrazgo Basin, a variety of groups of dinosaurs appear. Remains from a total of six styracosternan ornithopods (Gasulla et al., 2015; Medrano‐Aguado et al., 2023; Verdú et al., 2017), some sauropods (Gasca & Canudo, 2015; Mocho et al., 2017), theropods (Alonso & Canudo, 2015; Gasca et al., 2014b), and nodosaurian (Kirkland et al., 2013) have been found.

The fossil remains studied in this paper are from an ornithopod of the clade Iguanodontia, probably a styracosternan. This can be confirmed by the presence of an amphicoelous caudal vertebra, typical of this dinosaur clade (Norman, 1980, 1986). Moreover, the general morphology of the caudal vertebrae is similar to those assigned to the genus *Iguanodon* (Norman, 1980; Verdú et al., 2017). The vertebrae studied are very different from others assigned to theropods from the Barremian of the Iberian Peninsula, which are longer and have an hourglass or reel‐like shape (Malafaia et al., 2019); to sauropods, which are usually procoelous and have more circular articular surfaces (Royo‐Torres, 2009); or to the nodosaurids, which are lower in height and wider (Kirkland et al., 2013).

The centrum of the dorsal vertebra is heart‐shaped and amphicoelous, as in other styracosternan ornithopods (Gasulla et al., 2015). The dorsal vertebrae of theropods are usually longer than tall (Malafaia et al., 2019); those of sauropods are platycoelous and taller (Royo‐Torres, 2009); and in nodosaurids, the articular faces are circular (Vickaryous et al., 2004).

The ribs are bones of limited taxonomic value, although the general morphology of the ribs from Azud Aliaga is consistent with those described in styracosternan ornithopod dinosaurs (e.g., Bonsor et al., 2023; Medrano‐Aguado et al., 2023; Ruiz‐Omeñaca, 2011).

The ischium is very fragmentary but looks similar to ischia from other specimens of *Iguanodon* (Norman, 1980; Verdú et al., 2017). It is transversely flattened and shares with them a curved shaft that projects anteriorly. Other dinosaur groups can be ruled out, such as theropods, which have ventrally curved but otherwise straight ischial shafts (Lacerda et al., 2023), or ankylosaurs and sauropods, which have a more robust, dorsoventrally expanded ischium (Royo‐Torres, 2009; Vickaryous et al., 2004).

The tibia and the fibula of the Azud Aliaga specimen show similar morphologies to those of Early Cretaceous styracosternan ornithopod taxa closely related to *Iguanodon* (Gasca, 2011; Gasca et al., 2014a; Norman, 1986; Verdú et al., 2017). On the one hand, the general anatomical characteristics of these long bones do not allow a precise assignment within iguanodontian clades but differ from other, more slender ornithopods such as *Hypsilophodon* (Galton, 1974). On the other hand, with respect to the distal end of the tibia, MPZ 2024/96 can be distinguished from hadrosaurids by the reduced prominence of the malleoli (e.g., Godefroit et al., 2004: Figure 16).

### 4.2 Osteohistological description

To facilitate a reading of the description of each sample, a glossary of abbreviations for the histological structures is provided in Table 2.

**TABLE 2:** Palaeohistological nomenclature.

Histological structure	Abbreviation
Apposition front	AF
Haversian substitution front	HSF
Resorption front	RF
Primary osteons	PO
Secondary osteons	SO
Sharpey's fibres	SF
Lines of arrested growth	LAG
External fundamental system	EFS

#### 4.2.1 Dorsal ribs (MPZ 2024/92 and MPZ 2024/93)

The two ribs share a similar microstructure and development, although the distribution of the cancellous and compact tissue varies considerably between each sample. This difference in distribution is common in ribs of all types of vertebrates, even when relatively homologous cut points are selected (Canoville et al., 2016). The external morphology of each rib also varies, being similar at the proximal cut points, but different at the most distal ones. This difference in the distal sections could be due to a minor difference in the cutting point, or it could occur because their reniform morphology varies slightly depending on their more anterior or posterior location within the rib cage.

**FIGURE 3:**
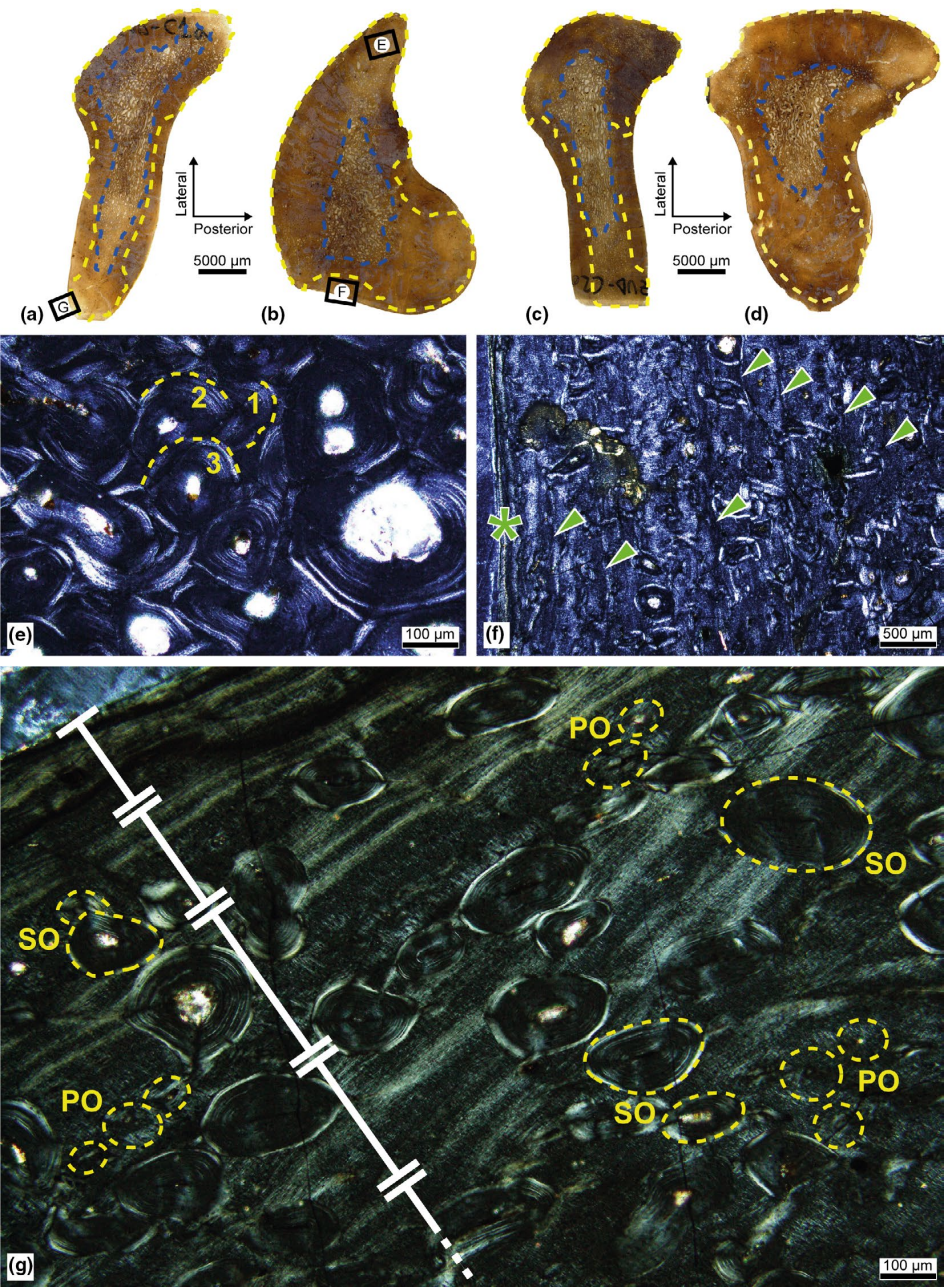
Iguanodontidae indet. from the Azud Aliaga site. Palaeohistological sections of the ribs. [a–d] Cross‐sections of the four rib samples: MPZ 2024/92‐L1 (a), MPZ 2024/92‐L2 (b), MPZ 2024/93‐L1 (c), and MPZ 2024/93‐L2 (d). Blue: Resorption front. Yellow: Haversian substitution front. (e) Magnification of the upper framed area in B under polarized light: Haversian tissue of the lateral area of MPZ 2024/92‐L2 with three generations of SO. (f) Magnification of the lower framed area in B under polarized light: Anteromedial area of MPZ 2024/92‐L2 with seven LAGs (green arrowheads) and the change of birefringence in the tissue (asterisk). (g) Magnification of the framed area in A under polarized light: Medial area of MPZ 2024/92‐L1 showing the change in birefringence between five intervals of LAGs (white lines). Yellow dashed circles indicate primary osteons (PO) and secondary osteons (SO).

In the proximal cross‐sections, the cortical region presents compact tissue of homogeneous thickness in all directions, whereas in the most distal cross‐sections, the AF varies, generating a greater thickness by comparison, especially in the posteromedial corner. The morphology of the medullary region also varies considerably in relation to the cut point, its RF and degree of porosity decreasing distally, although in both cases it maintains its maximum diameter in a lateromedial direction (Figure 3a–d).

In the four sections, the tissue of the medullary cavity exhibits numerous layers of lamellar organization lining the trabeculae, indicating a secondary origin with several episodes of remodelling. In the cortical region, woven‐parallel complexes have also been extensively remodelled, generating dense Haversian tissue in both the endosteal and periosteal regions. The HSF is more invasive in the medial and especially lateral planes, with at least three generations of SO (Figure 3e). In the posterior and anterior areas, some segments of primary woven tissue are preserved; these are composed mainly of primarily longitudinally oriented canals organized in circumferentially oriented bundles. The low degree of anastomosis between the canals and the scarcity of PO also suggest low bone vascularization. In these spaces, SF are also visible, although they are more abundant in the posterior area.

The two ribs present a similar number of LAGs, sometimes grouped as multiple growth mark complexes. Except in the case of MPZ 2024/93‐L2, whose highest concentration of LAGs is visible in the posterior area, the samples have the highest number of LAGs in the anteromedial area. Furthermore, the thin sections of the proximal points have a slightly lower number of LAGs—at least five in both samples—than the most distal cross‐sections—at least seven—(Figure 3f).

At the cortex, a striking pattern of diffuse bands of parallel‐fibred tissue with high birefringence and LAGs at each end is also generated, alternating with stripes of isotropic woven‐parallel tissue like those that constitute the rest of the bone (Figure 3g). The thickness and number of iterations of this pattern vary with the anatomical plane, up to five intervals—with five additional LAGs—being expressed in the segments with the greatest development of this pattern.

#### 4.2.2 Ischium (MPZ 2024/94)

**FIGURE 4:**
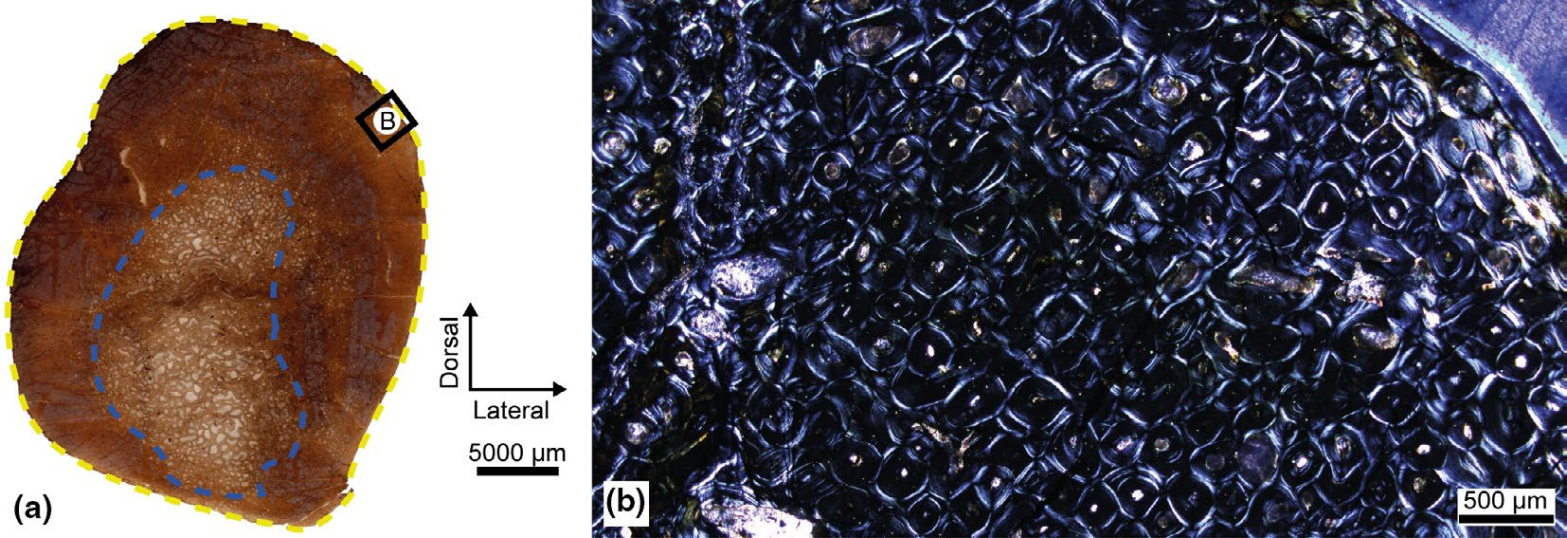
Iguanodontidae indet. from Azud Aliaga site. Palaeohistological section of the ischium. (a) Cross‐section of the ischium. Blue: Resorption front. Yellow: Haversian substitution front. (b) Magnification of the framed area in A under polarized light.

The ischium presents a medullary and a cortical region of similar proportions, except in the ventral area, where the RF generates a notable cortical narrowing. The transition between the two regions is relatively gradual, and the medullary cavity maintains a moderate degree of compaction, without large resorption chambers (Figure 4a).

The remodelling process was especially intense, generating dense Haversian tissue in the entire cortical region (Figure 4b). At least three generations of SO are identified, as are several resorption spaces that are slightly more porous compared to the adjacent compact tissue. Due to the intensity of the HSF, no further histological structures or growth marks are identified, apart from one multiple growth mark complex located at the cortex and partially obscured by remodelling.

#### 4.2.3 Fibula (MPZ 2024/95)

**FIGURE 5:**
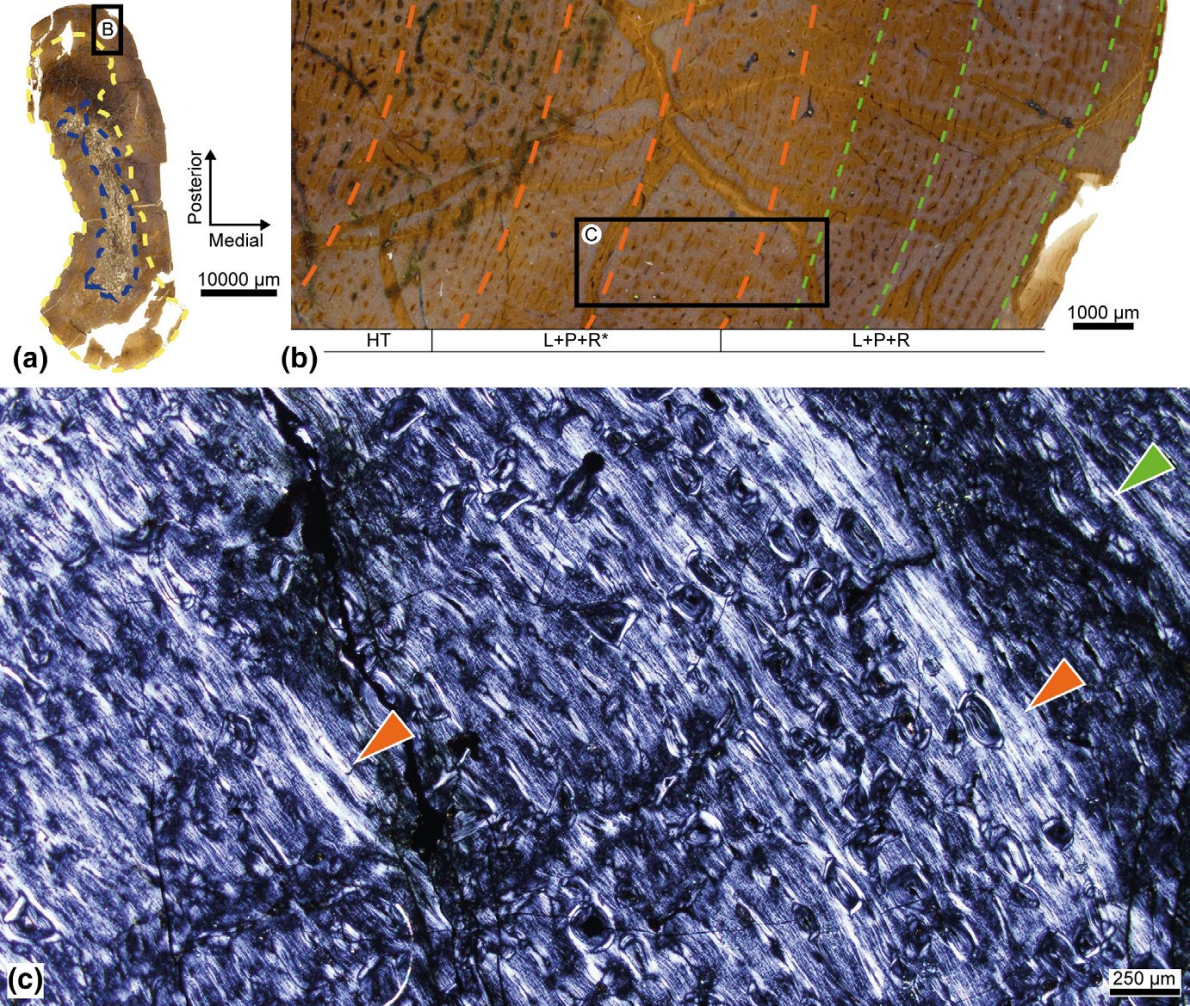
Iguanodontidae indet. from the Azud Aliaga site. Palaeohistological section of the fibula. (a) Cross‐section of the fibula. Blue: Resorption front. Yellow: Haversian substitution front. (b) Magnification of the framed area in A under normal light: The presence of *annuli* (orange) and LAGs (green) is indicated, as well as the distribution of vascular patterns. The first pattern indicated in every slot is the most abundant. The asterisk also indicates a very low presence compared to the others. HT, Haversian tissue; L, laminar; P, plexiform; R, reticular vascular patterns. (c) Magnification of the framed area in B under polarized light: Segment of the posterior area with development of *annuli* (orange arrowheads) and a LAG (green arrowhead).

In both its cortical and medullary regions, the diaphysis of the fibula shows a preferential expansion in a posteroanterior direction, implying a considerable thickening of the compact tissue in these areas. The medullary cavity is barely porous and establishes a very diffuse transition between cancellous and compact tissue (Figure 5a).

The HSF is uneven, varying with the anatomical plane. The lateral area develops dense Haversian tissue in its entire preserved region—it has lost a significant periosteal portion—whereas in the anterior area this tissue is also predominant towards the endosteal region. Towards the periosteal region, the presence of Haversian systems is less intense. Up to three generations of SO are identified.

The posterior and medial areas develop abundant Haversian systems in the endosteal region, which progressively reduce their presence towards the outside of the bone. In this direction, woven‐parallel complexes of variable extension abound. Simple canals are abundant, with poorly developed PO, but their arrangement generates different vascular patterns around the bone cortex.

In the poorly remodelled and woven‐parallel spaces of the anterior and medial areas, the vascular canals adopt a mostly plexiform pattern, whereas in the posterior area, they show greater variability. Laminar and reticular vascular patterns also emerge along with the plexiform arrangement in the parallel‐fibred framework. These seem to develop in line with the spaces delimited by growth marks (Figure 5b), and in both the posterior and anterior areas, low‐incidence SF are also identified.

In most primary spaces of the bone, growth marks are detected too, although their development is more striking in the posterior and posteromedial areas. In the inner periosteal region of the posterior area, four narrow stripes of greater birefringence are identified and seem to correspond to brief and cyclic slowdowns in the apposition and arrangement of the tissue, constituting *annuli* (Figure 5c), whereas in the outermost periosteal region, a total of four LAGs—sometimes grouped as multiple growth mark complexes—are generated.

#### 4.2.4 Tibia (MPZ 2024/96)

**FIGURE 6:**
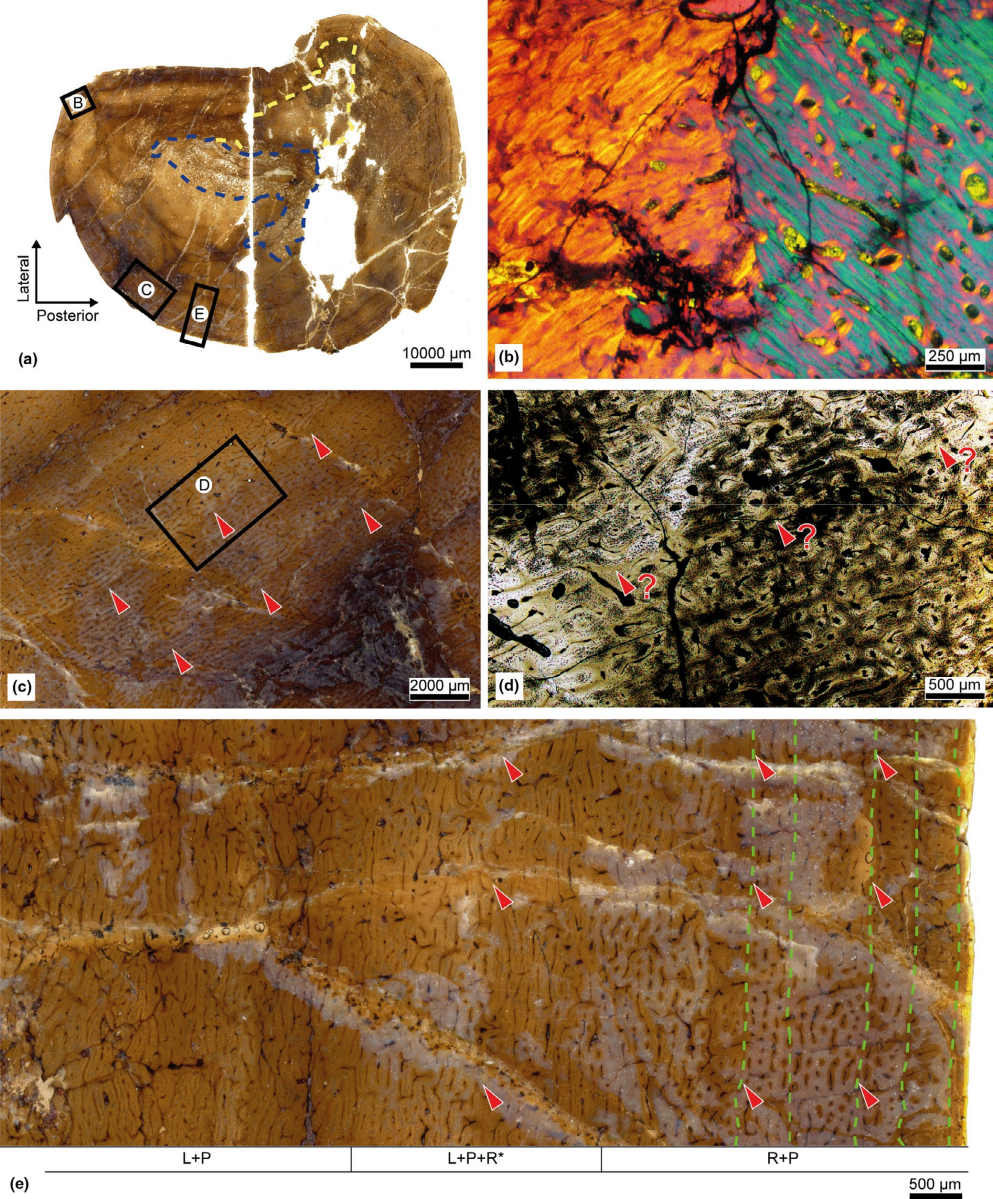
Iguanodontidae indet. from the Azud Aliaga site. Palaeohistological sections of the tibia. (a) Cross‐section of the tibia. Blue: Resorption front. Yellow: Haversian substitution front. (b) Magnification of the upper framed area in A under polarized light with a waveplate: The wedging of the tissue generates a different orientation of the collagen fibres on either side, easily discernible under polarized light. (c) Magnification of the lower left framed area in A under normal light: With the naked eye or in very low‐magnification digital scans, concentric marks can be detected around the bone (red arrowheads), although their thickness and number vary with the point observed. (d) Magnification of the framed area in C under normal light: On a micrometric scale, the delimitation of these concentric marks is not possible. (e) Magnification of the lower right framed area in A under normal light: The presence of LAGs (green) is indicated, as well as the distribution of vascular patterns. The first pattern indicated in every slot is the most abundant. The asterisk also indicates a very low presence compared to the others. L, Laminar; P, plexiform; R, reticular vascular patterns. Concentric marks are also indicated (red arrowheads), sometimes coinciding with changes in vascularization, sometimes with LAGs.

The diaphysis of the tibia has a high level of compaction, with a very large cortical region compared to the medullary region. The microanatomical preservation of the bone is impaired by fractures and the loss of some tissue fragments, making the delimitation of the medullary cavity imprecise. Likewise, its resorption chambers have a low degree of porosity, establishing a diffuse transition between spongy and compact tissue (Figure 6a).

By contrast with the rest of the samples analysed, the HSF of the tibia is restricted to a very localized section of the posterolateral area, producing up to three generations of SO that coincide with the presence of SF. The rest of the bone presents primary woven‐parallel complexes that alternates with a parallel‐fibred framework of greater birefringence towards the periosteal region. In the anterolateral corner, there is also a whirl‐like structure (Figure 6b), equivalent to the “anterolateral plug” described by Hübner (2012) in a tibia from a small ornithopod *Dysalotosaurus lettowvorbecki*, and later also identified in larger derived hadrosaurids (Freedman Fowler & Horner, 2015; Woodward et al., 2015).

Throughout the cortical region of the tibia, the proliferation of vascular canals and their degree of anastomosis vary markedly, with laminar and plexiform patterns being predominant in all anatomical areas. Furthermore, in the outermost periosteal region, the incorporation of a reticular type of pattern is frequent. On the other hand, these changes in vascularization sometimes coincide with the generation of concentric marks in the cortical tissue—up to a maximum of eight being identified in the anterior area—. These marks are observable only with the naked eye or in very low‐magnification digital scans (Figure 6c). Their optical properties are barely discernible at micrometric scale (Figure 6d), but those located on the periosteal edge are associated with LAGs or multiple growth mark complexes—up to five being counted—(Figure 6e). These segments show diffuse bands of parallel‐fibred tissue alternating with stripes of woven‐parallel tissue, similar to those described in the ribs.

## 5 DISCUSSION

### 5.1 Skeletochronology and ontogeny of the Azud Aliaga specimen

**FIGURE 7:**
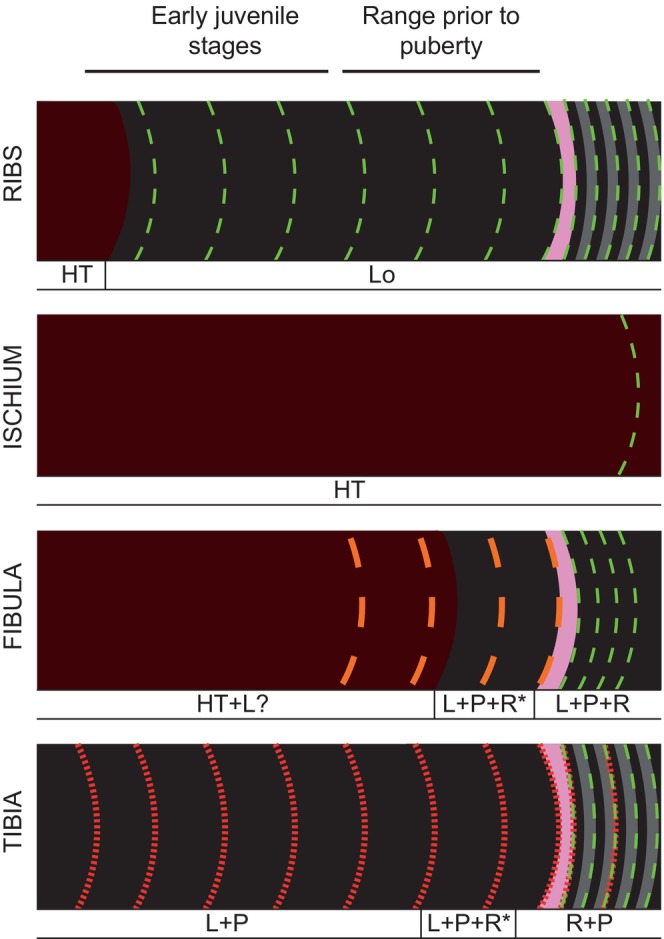
Formation of tissue throughout ontogeny according to the bone element: Growth marks include LAGs (green), *annuli* (orange) and concentric marks observable only with the naked eye or in very low‐magnification digital scans (red). The point of sexual maturity is indicated with a pink stripe, woven‐parallel complexes with a black background, Haversian tissue with a maroon background, and parallel‐fibred frameworks with a grey background. In multi‐pattern vascular slots, the first pattern indicated is the most abundant. The asterisk also indicates a very low presence compared to the others. It should be emphasized that this is a conceptualization of the overall distribution of histological structures in regions of preserved primary tissue and that their arrangement varies throughout the thin section. For example, a portion of the lateral area of the tibia displays only a predominantly laminar pattern, but overall it alternates according to the distribution shown in the figure. HT, Haversian tissue; L, laminar; Lo, longitudinal; P, plexiform; R, reticular vascular patterns.

For a skeletochronological and ontogenetic study, it is usual to select specimens with very complete skeletons, articulated and/or found in anatomical relationship. With material such as that described in this paper, some assumptions must be justified. Despite the fragmentary state of the samples and evidence of weathering, the original hypotheses of skeletal unity were based on observations regarding the size of the elements, their proximity in the field, and the lack of skeletal duplicities. After the histological review, the assumption that all the samples belong to the same individual is also supported by two histological markers (Mitchell et al., 2017; Wiersma‐Weyand et al., 2021): a similar rate of remodelling evidenced by up to three generations of SO in all the bones, and the correlation between the different types of growth marks (Figure 7).

The osteohistological analysis shows that the quantity and type of growth marks vary moderately between the ribs, fibula, and tibia, highlighting differences in the relative development of these elements as the specimen grew. Of these elements, the dorsal ribs have the best skeletochronological record in their anteromedial area. Table 3 provides a count of the different growth marks identified in each sample and the preferred anatomical area for their development or preservation.

**TABLE 3:** Record of growth marks on each sample.

Thin section ID (element)	Concentric marks	Annuli	LAGs	Preferred area
MPZ 2024/92‐L1 (dorsal rib)	0	0	10	Anteromedial
MPZ 2024/92‐L2 (dorsal rib)	0	0	12	Anteromedial
MPZ 2024/93‐L1 (dorsal rib)	0	0	10	Anteromedial
MPZ 2024/93‐L2 (dorsal rib)	0	0	12	Posterior
MPZ 2024/94‐L1 (ischium)	0	0	1	Dorsal?
MPZ 2024/95‐L1 (fibula)	0	4	4	Posterior
MPZ 2024/96‐L1 & MPZ 2024/96‐L2 (tibia)	8	0	5	Anterior

The vascular patterns show variability as well, depending on the skeletal element. Given their apparent relevance in the structures of bones such as the tibia, it seems advisable to discuss their different arrangement in each sample. In initial studies by Castanet et al. (1996), different vascular orientations appeared to be associated with specific apposition rates. In their study, the plexiform pattern is associated with a lower growth rate than the laminar and reticular patterns, the latter being associated with higher daily growth rates. On the other hand, the longitudinal and radial patterns involve a wider range of apposition (Castanet et al., 2000; Starck & Chinsamy, 2002), although a bone composed only of the longitudinal pattern shows the lowest growth rates (Castanet et al., 2000). However, subsequent quantitative analyses of these vascular orientations in relation to growth rate do not show a clear relationship between the two factors (de Buffrénil et al., 2021; de Margerie et al., 2002, 2004; Starck & Chinsamy, 2002). For this reason, in the current study, we report the orientations in evidence and assess their degree of similarity or dissimilarity, trying to determine the possible influence of an associated apposition rate or biomechanical factors.

#### 5.1.1 Ribs

The ribs show a high degree of remodelling related not only to the ontogenetic development of the individual but also to the strain frequency caused by the mechanics of breathing (Waskow & Sander, 2014). Primary tissue spaces preserve up to seven well‐spaced LAGs between segments of woven‐parallel tissue of low birefringence, reflecting little change in their speed of apposition and orientation as the bone increased in size. The spacing between these LAGs is initially homogeneous. However, starting from the seventh interval, a notable narrowing is seen between the most peripheral LAGs.

The LAGs along the cortex, numbering between two and five depending on the anatomical area, are associated with a birefringent pattern that could reflect some type of cyclical or seasonal slowdown in each annual interval. The isotropic iteration would thus correspond to a warm period with an abundance of resources, and the anisotropic one to a less benign period (Hübner, 2012; Klevezal, 1996). These peripheral LAGs also undergo a drastic reduction in spacing, indicating a critical decrease in tissue apposition that suggests the attainment of sexual maturity (Erickson et al., 2007). In total, between nine and twelve annual growth intervals were identified in all the samples, with a slowdown and possible sexual maturity appearing between the seventh and eighth intervals (see Ribs in Figure 7).

In terms of vascularization, these elements show poor transverse anastomosis and a longitudinal arrangement, suggesting an invariable metabolic rate throughout the individual's life cycle.

#### 5.1.2 Ischium

In the ischium, the only multiple growth mark complex identified at the cortex suggests that the general growth dynamics of this bone may have involved the production of LAGs, at least at the time of the specimen's death. Due to the intense remodelling, it is not possible to know at what point in the ontogeny the LAGs began to be generated, and it is unknown whether its vascularization was homogeneous as in the ribs or underwent some variability as in the long bones (see Ischium in Figure 7).

The reason for this intense remodelling is not clear. The cut point was chosen as close as possible to the ossification centre, which is located in the flattest section of the bone that is not preserved in our sample. This distance from the centre could imply a shorter skeletochronological record, but not necessarily greater remodelling since studies in avian dinosaurs have shown that this element does not develop further ossification centres (Hogg, 1980; Maxwell, 2007, 2008; Pourlis & Antonopoulos, 2013; Watanabe, 2017). However, ornithischians as a whole show divergent pelvic development compared to paravians and other archosaurs (Griffin et al., 2022), which could involve the osteogenesis of a second ossification centre that would have generated this intense remodelling in the area between the two centres. Whatever the case, this process shows interspecific variation in birds both for biomechanical factors and for reasons associated with precocial or altricial development (Maxwell, 2008; Pourlis & Antonopoulos, 2013; Watanabe, 2017). Given that this issue has been poorly studied in other dinosaurs, a more comprehensive study of the palaeohistology of the pelvis and its various skeletal elements in the fossil record may answer this question in the future.

Another possibility for this intense remodelling could be high biomechanical demands during locomotion, although no SF were identified to suggest a particularly incidental muscle insertion. If this possibility is correct, it would be consistent with the hypothesis of more complex locomotion in intermediate‐sized ornithopods compared to smaller ones, since in other pelvic bones such as the pubis it has been observed that small ornithopods show a lesser remodelled histology (Hübner, 2012).

#### 5.1.3 Fibula

In the fibula, the intercellular matrix contains a woven‐parallel arrangement. The presence of four *annuli* and later four LAGs suggests that the replacement of one type of growth mark by another could possibly be related to physiological slowdowns prior to puberty, which would not yet imply a total cessation of apposition at any time during each annual cycle. Upon reaching reproductive maturity, however, LAGs would begin to be generated with a closer spacing between them compared to the zones between the *annuli* (see Fibula in Figure 7). Furthermore, the birefringent alternation identified in the ribs and the tibia was not found in the fibula. This may be due to the lack of the periosteal border mentioned above, where this pattern is seen in the other bone elements. Another possibility would be that the fibula developed a different growth dynamic without this alternating birefringence, as *annulus* formation has not been identified in the other samples.

As regards vascularization, the fibula initially displays a plexiform pattern, to which laminar and reticular patterns are added at a more advanced ontogenetic stage. This addition of patterns prior to puberty suggests a change in bone physiology as the specimen grew, perhaps related to biomechanical factors (de Margerie et al., 2002).

#### 5.1.4 Tibia

The tibia only produces five LAGs on the outer cortex, also generating a birefringent pattern similar to that described in the ribs. It is remarkable that until this stage the bone does not produce LAGs or *annuli*, but on a macroscopic scale a series of concentric marks around the bone can be seen (up to a maximum of eight), corresponding sometimes to changes in vascularization. If these marks were counted together with the LAGs, 13 growth intervals would be represented, almost the same amount as are expressed in the ribs. However, given the under‐explored state of structures like these, which do not bear an exact resemblance to other unusual marks such as localized vascular changes (Woodward, 2019) or polish lines (Sander, 2000), this study chooses to count only the five identified LAGs as annual growth intervals. The nature of these changes in vascularization will require a larger and more complex study in the future, although Köhler et al. (2012) and Woodward et al. (2015) suggest that they may be associated with cyclic hormonal changes unrelated to annual events.

Considering the distribution of growth marks and birefringence changes in the other samples, the abrupt generation of LAGs and parallel‐fibred tissue in the outer cortex may also reflect sexual maturity (see Tibia in Figure 7). This growth dynamic would be like that identified in the hadrosaur *Maiasaura peeblesorum* by Woodward et al. (2015), in which the first LAG appears in individuals of considerable size, and which previously only showed modifications in the diameter and orientation of the vascular canals. Other structures such as the “anterolateral plug”, also present in the Azud Aliaga specimen, likewise appear developed in the larger individuals, but not in the smaller ones. On the other hand, the presence of these “plug structures” appears to show some variability with respect to early‐diverging taxa such as *Dysalotosaurus lettowvorbecki*, which develops them in late juvenile and sexually immature individuals as well (Hübner, 2012). Another possibility noted by Woodward et al. (2015) is that a cortical decrease in limb bones may reflect an ontogenetic transition from a bipedal to a quadrupedal posture. Such modifications associated with locomotion have also been recorded in the ornithischian ceratopsid *Psittacosaurus lujiatunensis* (Zhao et al., 2013). Nevertheless, given the lack of further appendicular bones in the Azud Aliaga specimen, this study cannot analyse this possibility at the current time.

As regards vascularization, the tibia alternates between laminar and plexiform patterns from its endosteal region outwards, with the reticular pattern also appearing in the periosteal region and becoming increasingly abundant in the cortex. As in the fibula, the addition of new patterns in the periosteal region seems to coincide with the attainment of more advanced ontogenetic stages.

#### 5.1.5 Overall ontogeny

Synthesizing this set of inferences, the specimen recovered at the Azud Aliaga site would have reached sexual maturity at around seven years of age and would have survived until an age of at least 9–12 years, without reaching skeletal maturity. At the time of its death, the ontogenetic stage of the specimen would be defined as a sexually mature, sub‐adult individual. It should be noted that this age range is approximate since the growth marks corresponding to earlier ontogenetic stages are obscured by the HSF and the RF.

Changes in the vascularization of the long bones seem to support this ontogenetic interpretation, as the addition of new patterns, especially the reticular pattern, is generated in accordance with the hypothetical attainment of sexual maturity. Given that the ribs do not show these modifications, it seems reasonable to assume that these changes, which are only seen in the limb bones, may be related to changes in locomotion or biomechanical factors, as suggested by de Margerie et al. (2002), rather than factors associated with the apposition rate, as observed by Castanet et al. (1996).

Overall, the history of the specimen's tissue development seems consistent with the “basal growth syndrome” proposed by Werning (2012), but the presence of a complex vascular system—as in the limb bones—and a high degree of remodelling appear to be typical of the “syndrome of extended rapid growth”. This mixture of the characteristics of both dynamics resembles other intermediate‐sized taxa such as the ornithopod *Tenontosaurus tilletti* (Werning, 2012) and the rhabdodontid *Rhabdodon* sp. (Prondvai, 2014), suggesting that the Azud Aliaga specimen would have grown much more slowly to adult size compared to larger members within Hadrosauridae (Werning, 2012).

The timing of sexual maturity is consistent with what has been observed in other ornithopods from the Iberian Peninsula, such as an indeterminate styracosternal iguanodontid (Maíllo et al., 2023) and *Draconyx loureiroi* (Waskow & Mateus, 2017), although the latter shows a slightly delayed range. *Dysalotosaurus lettowvorbecki* (Hübner, 2012) and *Probrachylophosaurus bergei* (Freedman Fowler & Horner, 2015) also show a similar range. However, ornithopods with very different sizes and growth strategies, such as *Maiasaura peeblesorum* (Woodward et al., 2015) and *Jeholosaurus shangyuanensis* (Han et al., 2020), exhibit much earlier ranges. This suggests that the age ranges for reaching puberty are even more complex than those related to growth rate and skeletal maturity. The strategy of late sexual maturity is probably associated with survival or intraspecific competition (Hübner, 2012). Nevertheless, any such inference would ideally be based on a larger sample of different ornithopods to compare their ranges of sexual maturity.

Another possibility that could explain this disparity in the attainment of sexual maturity between ornithopods is that in some of them the production of LAGs does not occur on an exactly annual time scale, as is common in reptiles (Castanet & Baez, 1991; Chinsamy, 2005; Peabody, 1961), and therefore the skeletochronological quantification would not be completely accurate. The formation of LAGs would thus be influenced by intraspecific variability or by external factors such as seasonal change, starvation or disease, as is the case in several mammals (Calderón et al., 2021; Chinsamy, 2005; Klevezal, 1996; Nacarino‐Meneses & Köhler, 2018). In the case of our specimen, its LAGs and multiple growth mark complexes were quantified on the basis of a possible two‐phase or poly‐phase annual rhythm, as inferred by Hübner (2012) in *Dysalotosaurus lettowvorbecki*, but such cyclicity would not begin until the specimen reached sexual maturity and expressed a birefringence pattern. At an earlier ontogenetic stage, the LAGs observed in the ribs do not express this pattern, and there is a homogeneous spacing between them, which would therefore correspond to a conventional annual rhythm.

This assumption could provide new insights into some hypotheses on tissue plasticity, such as the one proposed by Starck and Chinsamy (2002), perhaps suggesting that each bone element has a different tissue plasticity. Thus, ornithopods such as our specimen or others with a “basal growth syndrome” could manifest an intermediate competence between the high plasticity of mammals, able to stop their growth when conditions are not favourable, and the loss of this adaptation in other groups that do not develop LAGs or barely develop them, such as the ornithurine birds studied by Starck and Chinsamy (2002) or the deeply nested ornithopods with a “syndrome of extended rapid growth”. The precise dynamics of LAG formation and growth reconstructions will require detailed studies of more taxa from different classes, with a variety of growth strategies, diets, parental behaviour and habitats, as well as considerations of intraskeletal histovariability.

### 5.2 Allometric analysis based on the three‐front model

The three‐front model of Mitchell and Sander (2014) was initially devised to conceptualize the similarities and divergences between long bones of different sauropod taxa, analysing the interaction between three fronts: the apposition front, the Haversian substitution front, and the resorption front. According to the model, bone development—growth in diameter—occurs through the apposition of new bone on the periosteal surface, forming the apposition front. As new bone is deposited, the cortex is resorbed along the endosteal region—the resorption front—. Haversian substitution also begins in the inner cortex and moves outwards in a more or less well‐defined Haversian substitution front. The model assumes a constant Haversian substitution rate, whereas apposition and resorption decrease over time, so all the changes to microstructure and histology result from relative changes in the rates of apposition and resorption (Mitchell & Sander, 2014).

In addition, their progression at different speeds and their preferred direction of expansion can provide a developmental explanation for the histovariability between taxa from other non‐sauropod dinosaur groups (de Rooij et al., 2023), as well as the identification of multiple individuals from remains originally attributed to a single specimen (Konietzko‐Meier et al., 2024).

Konietzko‐Meier et al. (2024) include an additional front, the endosteal apposition front, which involves the generation of compact tissue in the medullary cavity. This front requires a slower HSF than the RF in order not to obscure the reversal lines generated during the cessation of osteoclast activity. Like the sauropods studied by Mitchell and Sander (2014), the Azud Aliaga ornithopod shows a high degree of remodelling that prevents the reversal lines from being seen in any of its bones. Even the barely remodelled tibia does not show this cementation structure. For this reason, the present study does not take into account this additional front, which seems to be more appropriate in taxa with tubular bones with a very thin cortex and a low degree of remodelling (Konietzko‐Meier et al., 2024).

Here, we argue for the validity of the model for analysing the intraskeletal variation of a single individual, with the intention not only of verifying the skeletochronological potential of each element but also of ascertaining how the divergences established between these elements can offer complementary information that enriches the final ontogenetic interpretation.

In this analysis, we also assume a constant HSF speed throughout the ontogeny of our specimen. This assumption is motivated by its consistent application not only in sauropods (Mitchell & Sander, 2014), but also in ceratopsid ornithischians (de Rooij et al., 2023) and in small diapsid reptiles (Konietzko‐Meier et al., 2024). The development of an equal number of SO generations in all bones, as well as the similar diameter of their osteons, also suggests that the speed of this front is relatively homogeneous, at least compared to the AF and its different vascular patterns depending on the rate of apposition (Castanet et al., 1996, 2000).

Furthermore, when applying this model, we consider it desirable to specify which anatomical area of the bone is being interpreted. In our intraskeletal analysis, this nuance does not particularly influence our results as we are dealing with different bone elements, but when comparing them with those described in other works, it seems imperative to select homologous areas, or at least to ascertain whether the fronts change between them (see the comparative analysis below).

#### 5.2.1 Three‐front model of the Azud Aliaga specimen

The ribs are heavily invaded by dense Haversian tissue, indicating the cessation of the AF and RF in the ontogenetic stage reached by the specimen. The posterior and anterior areas are less affected by the HSF, although the proportion of primary tissue preserved varies with the cross‐sectional height. In the proximal sections, under the tuberculum, the posterior and anterior areas show a similar extent of primary tissue, greater than in the most distal sections, but with fewer LAGs. The most distal sections present a greater amount of primary tissue in the posterior area than in the anterior, but despite this, the more restricted spaces in the anterior area preserve a greater number of LAGs.

The fronts of the ischium reflect the same range of speeds as in the ribs at this ontogenetic stage, but its development is more homogeneous in all directions and there is no preserved primary tissue. Analysis of the proportion of compact tissue and the expansion of the medullary cavity nevertheless reveals that the speed of the RF is higher than that of the AF in the ventral area, with the inverse relationship occurring in the dorsal area.

In the fibula, the speeds of the AF and RF vary with the anatomical area. In the ontogenetic stage reached by the specimen, the RF has ceased completely in the lateral and anterior areas, allowing the dense Haversian tissue to surpass the AF. The posterior and medial areas present a faster AF compared to the HSF, thus preserving a moderate portion of primary tissue. The lateromedial narrowing of the medullary cavity also indicates that the AF is greater than the RF in that direction.

The tibia is the only element that shows in almost all its anatomical areas a higher‐speed RF compared to the HSF, as well as an AF that is considerably more developed than the other two fronts in any area of the bone.

**FIGURE 8:**
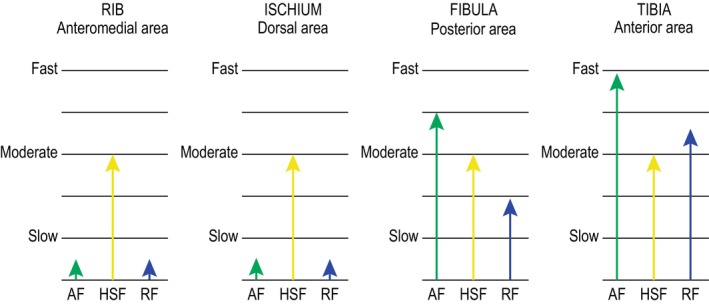
Three‐front models of each bone. The cessation of the AF and RF of the flat bones stands out in relation to the long bones, which maintain an active AF and RF at different speeds.

Comparing the models of all the samples (Figure 8), the three fronts of each bone element show variable speeds, although a primary clustering can be established that differentiates the long bones—fibula and tibia—from the flat ones—ribs and ischium—. This distinction may be due to the different metabolic requirements of each group of elements. The ribs and ischium would thus be bones that start remodelling at a higher intensity to limit the transfer of nutrients through the bloodstream—and thus maintain a specific size and morphology—whereas the long bones would not undergo such invasive remodelling and could maintain an AF with a faster speed (Padian et al., 2015).

In addition to the fact that each cluster shows a very different progression of speed ranges, the symmetry or asymmetry of the fronts in the different anatomical areas is also striking. The long bones have fronts that develop unequally depending on the anatomical area (see Figure 5a and Figure 6a), whereas the flat bones show a relatively equal distribution (see Figure 3a–d and Figure 4a). This development contrasts with the morphological symmetry of each cluster. In sum, the long bones show a symmetrical morphology but with an asymmetrical development of their fronts, whereas the flat bones show the converse relationship. Future studies increasing the number and type of elements may help to clarify whether this relationship is causal or whether it varies without specific functionality, as all the other work using this model to date has focused only on highly symmetrical appendicular bones.

Finally, we would like to point out that in order to better understand an individual's tissue development and avoid diagnostic errors, as many elements as possible should be analysed together. Taken in isolation, the ribs and ischium suggest that the individual would have been at a very advanced ontogenetic stage, but this contrasts with our final result. Similarly, the mismatch in AF and RF between the long bones could have led to the erroneous hypothesis that they belonged to two different individuals, the tibia being assigned to a slightly younger individual than that inferred from the fibula. A combined analysis of tissue fronts, correlation of growth marks, and the remodelling rate is therefore essential to avoid potential misinterpretations when applying this model, at least when comparing different bone types.

#### 5.2.2 Comparison with three‐front models in other studies

Since its conception, the three‐front model has been referenced many times, although its systematic application has been limited to just a few fully represented examples and has focused exclusively on limb bones. Mitchell and Sander (2014) applied it to the humeri and femurs of *Apatosaurus*, *Giraffatitan*, *Camarasaurus*, an undetermined diplodocid from Tendaguru, *Europasaurus*, *Magyarosaurus*, and *Ampelosaurus*. Among ceratopsids, de Rooij et al. (2023) extended the record to several limb bones of *Triceratops*, notably the autopodial and zeugopodial forelimb—radius, ulna, and humerus—and hindlimb—fibula, tibia, and femur. Finally, Konietzko‐Meier et al. (2024) applied the model to the humerus and femur of the small diapsid reptile *Ozimek volans*. In this section, we summarize the results of these studies, incorporating data from our ornithopod specimen where possible.

The speed ranges of the fronts show relatively homogeneous dynamics among the different taxa groups, although some differences are noted. The definition of histological ontogenetic stages—HOS—in sauropods by Klein and Sander (2008) allowed Mitchell and Sander (2014) to show that, in their specimens, the AF and RF slow down during sexual maturity—at HOS 8—and are finally overtaken by HSF when skeletal maturity is reached—at HOS 12—. In *Triceratops*, de Rooij et al. (2023) note a greater allometry between its elements and the ontogenetic development. There is a reduction in the AF and RF speeds between the youngest and oldest individuals, but in the radius and ulna the AF speed remains higher than the HSF in both cases. With respect to *Ozimek volans*, Konietzko‐Meier et al. (2024) note that this taxon does not develop an HSF, but that the AF and RF are also reduced during ontogeny. Overall, in all the taxa the AF is always higher than the RF in the earliest stages, whereas in advanced stages they may be equal in some skeletal elements. Only in the adult stage of *Europasaurus* is the RF slightly higher than the AF, although they eventually become equal during senescence.

In our study, we see that in limb bones a lower cortical thickness implies a lower RF speed and a higher degree of remodelling (compare the RF speed between the tibia and the fibula in Figure 8 and the cortical thickness between Figure 5a and Figure 6a). This relationship is also found in *Triceratops* by de Rooij et al. (2023), and between the femur and humerus of *Ozimek volans* (Konietzko‐Meier et al., 2024). As regards the ribs, de Rooij et al. (2023) include this element in their histological analysis, but not in their three‐front model. Nevertheless, from their histological description, it can be inferred that the ribs of *Triceratops* develop tissue fronts like those observed in the Azud Aliaga ornithopod.

The dataset not only confirms high allometry between the different appendicular elements but also identifies common dynamics, such as the range of slowing of each front or the relationship between cortical thickness and the greatest remodelling in limb bones. Of the non‐appendicular bones, ribs are the only element that could be taken to show the same dynamics in their tissue fronts, but it should be borne in mind that the degree of remodelling is highly variable along the rib cage (Waskow & Sander, 2014). This relationship could be coincidental, or biomechanical factors could be more influential. In terms of phylogenetic implications, Mitchell and Sander (2014) found that some sauropod taxa of similar size have identical tissue fronts at all ontogenetic stages, in contrast to smaller ones. Whether this common histology also emerges among other non‐sauropod groups will require further study using the three‐front model.

### 5.3 Areas with greatest skeletochronological potential and comparison with other ornithopods

Our analysis of how the apposition, resorption, and remodelling fronts interplay in each bone allows us to infer which segments of the anatomical plane are best suited to provide palaeohistological information related to skeletochronology. Additionally, this information is also valuable during the preparation of a thin section, not only in the case of incomplete bone elements but also for those that require the application of core drilling (Stein & Sander, 2009).

Here, the areas of each bone with the greatest potential to preserve primary tissue are evaluated. Likewise, a comparison is made with equivalent bone elements from a wide variety of ornithopods studied by other authors, ranging from early‐diverging taxa to deeply nested ones. In this selection, we attempt to compare the different ornithopods by considering histological properties—remodelling rates, vascular patterns and collagen fibre arrangement—that have developed by an adult ontogenetic stage, or at least by a late juvenile stage.

#### 5.3.1 Ribs

In the case of the dorsal ribs from the Azud Aliaga specimen, the posterior and anterior areas preserve the greatest amount of unremodelled primary tissue, and contrary to the development suggested by Waskow and Sander (2014), more distal samples contain more LAGs than proximal ones. The number of preserved growth marks increases slightly towards the medial and especially anteromedial areas. These results agree partially with those observed in the early‐diverging *Draconyx loureiroi* (Waskow & Mateus, 2017), whose largest record of growth marks was preserved in the posteromedial segment, but whose cortical tissue in general showed almost no remodelling. By contrast, other deeply nested taxa of large‐sized ornithopods such as *Saurolophus angustirostris* and *Barsboldia sicinskii* show strongly remodelled ribs (Słowiak et al., 2020).

Moreover, all four taxa orient their vascular canals longitudinally, although *Saurolophus angustirostris* also develops a reticular pattern in the proximal sections of the largest individual. Overall, this osseous element shows a homogeneous development of vascularization, but great variability in remodelling. These differences could be due to a different distribution in the rib cage, or to the fact that the sections are not exactly homologous in the proximal‐distal axis.

In terms of the collagen fibre arrangement, *Draconyx loureiroi* shows an intercellular matrix similar to that of the Azud Aliaga specimen, with more slowly growing lamellar‐zonal bone towards the bone surface. *Saurolophus angustirostris* and *Barsboldia sicinskii*, on the other hand, show a purely woven‐parallel complexes in their cortical tissue.

In summary, in the Azud Aliaga ornithopod, the rib is the element that preserves the greatest amount of growth marks, and its anteromedial and more distal areas provide the best skeletochronological record compared to other bone elements.

#### 5.3.2 Ischium

In the ischium, the advancement of the HSF has obscured the preservation of growth marks, but the evolution of the AF seems to suggest that, at earlier ontogenetic stages in which the remodelling is not yet so invasive, the dorsolateral area would have been the point most susceptible to developing skeletochronological markers. No recently studied taxa have been found that have included this element, which would allow a broader record to be considered.

In our study, this element proved to be irrelevant from a skeletochronological point of view, at least in the anatomical section studied. Without further research on the centres of ossification in ornithopod dinosaurs, or the influence of biomechanics on their remodelling, it is not possible to say at present whether its value might be different in other more proximal or distal sections of the bone.

#### 5.3.3 Fibula

The fibula from the Azud Aliaga specimen shows a preferential remodelling of the lateral and anterior areas, so the medial and especially posterior areas are those most likely to preserve most palaeohistological information. This distribution seems to vary notably among ornithopods. The remodelling in *Jeholosaurus shangyuanensis* does not go beyond the endosteal region (Han et al., 2020), whereas *Dysalotosaurus lettowvorbecki* presents a more invasive HSF in the medial area (Hübner, 2012). *Tenontosaurus tilletti* develops intense remodelling even in the periosteal region (Werning, 2012). As regards derived ornithopods such as *Saurolophus angustirostris*, their HSF is concentrated in the endosteal region (Słowiak et al., 2020).

Although the early‐diverging *Jeholosaurus shangyuanensis* and *Tenontosaurus tilletti* show a few radial and reticular patterns in their inner cortex, the orientation of the vascular canals is mainly longitudinal in all the taxa mentioned, regardless of their body size or phylogenetic relationships. Strikingly, only the specimen from Azud Aliaga does not show this arrangement of vascularization. The possible reasons for this are currently unknown.

With respect to the arrangement of collagen fibres, all the taxa exhibit dominant woven‐parallel complexes with outlying parallel‐fibred frameworks, except for the deeply nested *Saurolophus angustirostris*, which develops only woven‐parallel tissue.

As regards the fibula's skeletochronological potential, although this element in the Azud Aliaga specimen shows moderate remodelling and does not preserve the largest record of growth marks, the variety of types and arrangement of vascular patterns in the posterior area provide complementary information that enhances the ontogenetic interpretation.

#### 5.3.4 Tibia

As for the tibia, the Azud Aliaga specimen reveals an isolated remodelling episode in the posterolateral area that differs slightly from that observed in the tibias of *Dysalotosaurus lettowvorbecki* (Hübner, 2012), *Maiasaura peeblesorum* (Woodward et al., 2015), and *Probrachylophosaurus bergei* (Freedman Fowler & Horner, 2015), whose HSFs are concentrated in the endosteal region of the “anterolateral plug”. In the case of the Azud Aliaga specimen, the area of posterolateral remodelling extends almost to the outer cortex and coincides with the contact area of the fibula, suggesting a biomechanical requirement for remodelling related to the interaction between the two elements, or possibly with the tendonous insertion near the tibial border (Woodward et al., 2015). This type of hyperlocalized remodelling has also been identified in *Jeholosaurus shangyuanensis* (Han et al., 2020). By contrast, the early‐diverging *Mochlodon vorosi* and *Mochlodon suessi* show an HSF that invades most of the cortical tissue (Prondvai, 2014). *Tenontosaurus tilletti*, on the other hand, shows more moderate remodelling (Werning, 2012). As regards the deeply nested ornithopods, *Gobihadros mongoliensis* shows highly remodelled cortical tissue, whereas *Saurolophus angustirostris* barely undergoes this process (Słowiak et al., 2020).

In the Azud Aliaga specimen, the distribution of the RF and the ratio between the compact tissue and the reduced medullary cavity is also striking and looks similar to *Probrachylophosaurus bergei*. Compared to other ornithopods of varying sizes, such as *Jeholosaurus shangyuanensis*, *Tenontosaurus tilletti*, and *Maiasaura peeblesorum*, there is a qualitative difference even in relation to adult specimens of these taxa, which have already experienced a significant reduction in their cancellous tissue. The cause of this discrepancy is unclear, as recent studies have ruled out osteosclerosis in most dinosaur clades other than theropods (Fabbri et al., 2022), although adaptations associated with aquatic biotopes have been suggested in some groups of ornithischian protoceratopoids and psittacosaurids (Ford et al., 2007; Tereschenko, 2008).

Of all the bones analysed, the tibia shows the most complex vascular development, with a dominant pattern in some taxa and an alternating pattern in others. The early‐diverging *Mochlodon vorosi*, *Mochlodon suessi* and *Jeholosaurus shangyuanensis* develop a longitudinal pattern in most of their bone tissue. *Tenontosaurus tilletti* has not only a longitudinal pattern in its outer cortex but also other less common patterns in the inner cortex. *Dysalotosaurus lettowvorbecki* develops only a laminar pattern.

As regards the deeply nested taxa, *Probrachylophosaurus bergei* shows a predominantly laminar pattern, and *Saurolophus angustirostris* develops an alternation between laminar and longitudinal tissue in the outer cortex, together with a reticular pattern in the inner cortex. Another deeply nested ornithopod, *Maiasaura peeblesorum*, also exhibits complex vascularization, including longitudinal, reticular, laminar and plexiform patterns.

In the tibia, the Azud Aliaga specimen suggests a physiology closer to that of deeply nested ornithopods, as it mainly alternates between plexiform and laminar patterns. On the other hand, as noted by Werning (2012), the early‐diverging ornithopods seem to display simpler vascularization than the deeply nested ornithopods.

In terms of tissue management, the observations of Werning (2012) also apply. Early‐diverging ornithopods and those with intermediate phylogenies develop woven‐parallel complexes and parallel‐fibred frameworks in the outer cortex. In contrast, deeply nested ornithopods develop only woven‐parallel tissue.

As far as skeletochronological potential is concerned, the tibia, like the fibula, retains a sparse record of annual growth cycles, but the vascular complexity and unusual structures identified concentrically around the bone provide unique information to be borne in mind in considering the overall ontogeny.

## 6 CONCLUSIONS

Osteohistological analysis has shown that the Azud Aliaga specimen attributed here to Iguanodontia indet. would have reached an age of at least 9–12 years at its death, reaching puberty at around the age of seven. At the given ontogenetic stage—individual subadult, sexually mature—, a certain variability has been identified in the type of growth marks expressed in each bone—LAGs, *annuli* and vascular changes with anomalous optical properties—, as well as alterations in the organization and birefringence of the tissue. Despite these divergences between elements, a progressive slowdown in tissue apposition can be discern, as well as a complex vascular system and a high degree of remodelling in some bones. These properties suggest that the Azud Aliaga specimen is consistent partially with the “basal growth syndrome” of Werning (2012), although at the same time histological markers associated with deeply nested taxa are also present. Of the four bone types analysed, those with negative allometry, the dorsal ribs, have the most homogeneous record and are the most suitable for skeletochronological interpretation, although the fibula and tibia provide complementary and unique information about each element that should not be ignored. Likewise, the application of the three‐front model to different bones from a single specimen has also made it possible to determine which anatomical areas show the greatest potential to preserve a primary palaeohistological record. However, comparison with other ornithopods reveals the great variability that each bone element shows depending on the taxon analysed, varying even between taxa of similar size or phylogenetic proximity. In the future, more detailed analysis of different bone elements and ornithopod taxa will clarify which bone element is most suitable for studying each taxon, as well as what type of growth dynamics can be established on the basis of their phylogenetic relationships.

## AUTHOR CONTRIBUTIONS

**Juan Maíllo:** Conceptualization, Methodology, Investigation, Visualization, Writing Original draft, Writing–review and editing. **Jerome Hidalgo‐Sanz:** Investigation, Visualization, Writing Original draft, Writing–review and editing. **José Manuel Gasca:** Conceptualization, Investigation, Writing Original draft, Writing–review and editing. **José Ignacio Canudo**: Investigation, Writing–review and editing. **Miguel Moreno‐Azanza:** Conceptualization, Methodology, Writing–review and editing, Supervision, Project administration.

## FUNDING INFORMATION

This work was supported by projects PID2021‐122612OB‐I00, MINECO/FEDER, UE, and RYC2021‐034473‐I, funded by MCIN/AEI/10.13039/501100011033, the European Union (NextGenerationEU), Gobierno de Aragón, ERDF (E18_23R Aragosaurus: Recursos geológicos y Paleoambientes), and the Fundação para a Ciência e Tecnologia (FCT‐MCTES) of Portugal (project PTDC/CTA‐PAL/2217/2021 and Research Unit GeoBioTec UIDB/04035/2020).

## Data Availability

The specimen and thin sections are housed in the Museo de Ciencias Naturales de la Universidad de Zaragoza. High‐resolution scans and images can be obtained by contacting the authors by email.
